# Echocardiographic, Biochemical, and Electrocardiographic Correlates Associated With Progressive Pulmonary Arterial Hypertension

**DOI:** 10.3389/fcvm.2021.705666

**Published:** 2021-07-20

**Authors:** Ahmed Zaky, Iram Zafar, Juan Xavier Masjoan-Juncos, Maroof Husain, Nithya Mariappan, Charity J. Morgan, Tariq Hamid, Michael A. Frölich, Shama Ahmad, Aftab Ahmad

**Affiliations:** ^1^Department of Anesthesiology and Perioperative Medicine, University of Alabama at Birmingham, Birmingham, AL, United States; ^2^Department of Biostatistics, University of Alabama at Birmingham, Birmingham, AL, United States; ^3^Division of Cardiovascular Disease, Department of Medicine, University of Alabama at Birmingham, Birmingham, AL, United States

**Keywords:** pulmonary arterial hypertension, echocardiography, disease progression, electrocardiography, SU5416, cardiac troponin T, cardiac troponin I, FABP-3

## Abstract

**Background:** Pulmonary arterial hypertension (PAH) is a progressive proliferative vasculopathy associated with mechanical and electrical changes, culminating in increased vascular resistance, right ventricular (RV) failure, and death. With a main focus on invasive tools, there has been an underutilization of echocardiography, electrocardiography, and biomarkers to non-invasively assess the changes in myocardial and pulmonary vascular structure and function during the course of PAH.

**Methods:** A SU5416-hypoxia rat model was used for inducing PAH. Biventricular functions were measured using transthoracic two-dimensional (2D) echocardiography/Doppler (echo/Doppler) at disease onset (0 week), during progression (3 weeks), and establishment (5 weeks). Similarly, electrocardiography was performed at 0, 3, and 5 weeks. Invasive hemodynamic measurements and markers of cardiac injury in plasma were assessed at 0, 3, and 5 weeks.

**Results:** Increased RV systolic pressure (RVSP) and rate of isovolumic pressure rise and decline were observed at 0, 3, and 5 weeks in PAH animals. EKG showed a steady increase in QT-interval with progression of PAH, whereas P-wave height and RS width were increased only during the initial stages of PAH progression. Echocardiographic markers of PAH progression and severity were also identified. Three echocardiographic patterns were observed: a steady pattern (0–5 weeks) in which echo parameter changed progressively with severity [inferior vena cava (IVC) expiratory diameter and pulmonary artery acceleration time (PAAT)], an early pattern (0–3 weeks) where there is an early change in parameters [RV fractional area change (RV-FAC), transmitral flow, left ventricle (LV) output, estimated mean PA pressure, RV performance index, and LV systolic eccentricity index], and a late pattern (3–5 weeks) in which there is only a late rise at advanced stages of PAH (LV diastolic eccentricity index). RVSP correlated with PAAT, PAAT/PA ejection times, IVC diameters, RV-FAC, tricuspid systolic excursion, LV systolic eccentricity and output, and transmitral flow. Plasma myosin light chain (Myl-3) and cardiac troponin I (cTnI) increased progressively across the three time points. Cardiac troponin T (cTnT) and fatty acid-binding protein-3 (FABP-3) were significantly elevated only at the 5-week time point.

**Conclusion:** Distinct electrocardiographic and echocardiographic patterns along with plasma biomarkers were identified as useful non-invasive tools for monitoring PAH progression.

## Introduction

Pulmonary arterial hypertension (PAH) is a progressive proliferative vasculopathy affecting small pulmonary arterioles culminating in increased vascular resistance and right ventricular afterload (1). According to a recent task force report, assessing RV function is an ongoing challenge (2).

Failure of the RV to adapt to increased afterload is the principal cause of death in patients with pulmonary hypertension (PH) (3, 4). Factors reflecting RV dysfunction by cardiac catheterization such as cardiac index and mean right atrial pressure are significant predictors of survival in patients with PAH (3). Additionally, a failing RV causes poor prognosis even if pulmonary vascular resistance is reduced (5), demonstrating the importance of evaluating and maintaining RV function in PAH patients.

Despite the prognostic significance of the RV status in PH, gaps still remain in the assessment of RV function and structure both during the course of the disease and during treatment (6). This stems in part from the lack of well-established clinical determinants of RV function, and the complex structure and orientation of the RV in the anterior chest that hampers a straightforward assessment using conventional imaging modalities (2, 7). Previous attempts to evaluate progression of PAH in animal models were limited in that invasive measurements were carried out at one time point with an underutilization of echocardiography to assess the pulmonary vasculature and the left ventricle during the course of PAH (6). While advanced imaging techniques can provide a better assessment of RV function, a vast number of clinicians still rely on conventional imaging modalities. Furthermore, less attention has been given to the assessment of the electrical function and trend of biomarker progression during the course of PAH. Understanding the biochemical, electrocardiographic, and echocardiographic patterns during the course of PAH may guide clinical management of patients with PAH and help identify patients at an early stage of the disease when therapies could potentially be more effective (8). Furthermore, recent change in the clinical diagnosis of PAH using a threshold of mean pulmonary artery pressure (mPAP) from >25 to >20 mmHg underscores the efforts in diagnosing PAH at an early stage (9, 10).

To address these gaps, we sought to perform a comprehensive analysis to assess electrical, biochemical, and mechanical changes that occur in the heart and in the pulmonary circulation during the progression of PAH in a SU5416-hypoxia rat model. Our objective is to identify sensitive indices that can be obtained and monitored non-invasively in the early diagnosis of PAH and during the course of PAH.

## Methods

### Animal Model of Pulmonary Arterial Hypertension

All animal experiments were performed under the University of Alabama Institutional Animal Care and Use Committee approval and in accordance with the National Institutes of Health Guide for the care and use of laboratory animals. This manuscript adheres to the ARRIVE guidelines. PAH was induced in rats using an established model (11). Briefly, adult male Sprague–Dawley rats weighing 160–200 g were injected subcutaneously with SU-5416 (20 mg/kg), a vascular endothelial growth factor receptor 2 (VEGFR-2) inhibitor, and exposed to normobaric hypoxia (10% O_2_) for 3 weeks (SuHyx rats). They were then returned to normoxia (21% O_2_, room air) for two additional weeks. For invasive measurements, separate sets of animals were used for the control, 3- and 5-week time points groups. For echocardiographic measurements, the same animals were used for the control (0 week), 3- and 5-week measurements. The number of animals used in each measurement is indicated in the figure legends. Experiments in each group were carried out independently for a minimum of two times.

### Hemodynamic Measurements

Hemodynamic measurements were performed in rats under 2% isoflurane anesthesia using a 1.4 F high-fidelity Millar catheter as described by us before (12). Using a Biopac data acquisition system and AcqKnowledge III software (ACQ 3.2), the rate of rise of ventricular pressure during systole (dP/dT_maximum_) and subsequent fall during diastole (dP/dT_minimum_) were measured. Systemic blood pressure was also monitored using the same catheter inserted in the carotid artery. In animals where echocardiography was carried out, invasive measurements were performed at the end of the 5-week protocol before the animals (*n* = 10) were euthanized. Separate sets of animals were used for invasive measurements of naïve (*n* = 17) and 3-week exposed animals (*n* = 11).

### Immunofluorescence Staining

Animals were euthanatized, and the left lung was inflation fixed with low-melting agarose and immersed in a solution of 10% formalin in ethanol for up to 48 h. The tissues were then processed for paraffin embedding. Five-micrometer-thick sections were cut on positively charged slides, and deparaffinization and antigen retrieval was performed. Sections were then blocked in 5% normal goat serum and incubated overnight with anti-von Willebrand factor (vWF) antibody (Dako cat# A0082) and anti-alpha smooth muscle actin (α-SMA) (Abcam cat# 18147). After washing with TBST (Tris-buffered saline with 0.025% Triton-X100), fluorescence tagged secondary antibodies, anti-rabbit Alexa fluor 488 (vWF), and anti-mouse Alexa fluor 594 (α-SMA) were applied, and sections were incubated for 1 h. Sections were then washed, rinsed with PBS, and mounted with VECTASHIELD containing DAPI (Vector laboratories). Images were captured at ×20 using the BZ-X800 Keyence microscope.

### Cardiac Biomarker Measurements

Levels of cardiac and skeletal muscle markers of injury were measured in the plasma of rats from 0-, 3-, and 5-week time points using the meso scale discovery (Rockville, MD, USA) muscle injury panel 1 kit. A multiplex assay to quantitate plasma levels of cTnI (cardiac troponin I), cTnT (cardiac troponin T), FABP3 (fatty acid-binding protein 3), Myl3 (myosin light chain 3), and sTnI (skeletal troponin I) were carried out using standards and as per protocol of the manufacturer.

### Echocardiography and Electrocardiography

Transthoracic echocardiography and electrocardiography were performed in anesthetized animals (2% isoflurane) as described by us before (12). Echocardiography was performed prior to, at 3 and 5 weeks post exposure using a Vevo2100 high-resolution ultrasound system (Visual Sonics Inc., Toronto, ON, Canada) using a 13- to 24-MHz linear transducer (MS-250). Rats were placed supine on the warmed stage (37°C) of the echocardiography system. Two-dimensional cardiac images were acquired from the parasternal long- and short-axis, apical, subcostal, and suprasternal views using M-mode and B-modes at mid papillary level and averaged to determine the RV and LV dimensions at end systole and end diastole as described (13).

The RV and LV volumes, cardiac output, fractional shortening, fractional area of change, and ejection fraction were obtained according to guidelines (14). The LV systolic and diastolic eccentricity index was calculated as the ratio of the LV anteroposterior dimension and the septolateral dimension. The parasternal pulmonary artery view was obtained, and pulsed wave Doppler was used to measure flow across the RV outflow tract. End systolic diameter of the pulmonary artery was measured, and the end systolic diameter of the pulmonary artery to the end systolic diameter of ascending aorta ratio (stiffness index) was calculated. Apical four-chamber views with B- and M-modes were obtained to determine tricuspid annular plane systolic excursion (TAPSE). Pulsed wave Doppler was used to determine transmitral and transtricuspid early (*E*) and atrial (*A*) wave peak velocities, isovolumic relaxation time (IVRT), E-wave deceleration time, and isovolumic contraction time, with the ratio of *E* to *A* calculated across both the mitral and tricuspid valves. A tricuspid regurgitant jet was sought to estimate the RVSP when discernable (15). Tissue Doppler imaging was used to determine lateral mitral and tricuspid annular diastolic peak early (*E*′), late atrial (*A*′), systolic (S′) annular velocities, and (*E)* to *(E*′*)* ratios were calculated. Myocardial performance index (MPI) for both ventricles was calculated from the spectral Doppler tracing of transmitral and transtricuspid flows as described (16). A subcostal inferior vena caval view was obtained, and the inferior vena caval diameter was measured at end inspiration and end exhalation. Pulsed wave Doppler was used to assess hepatic venous blood flow.

For electrocardiography, a two-channel electrocardiography was performed on anesthetized rats prior to, at 3 and 5 weeks post exposure. To obtain ECG tracings, bipolar platinum electrodes were positioned in the thorax (subcutaneous tissue) directly in derivation DII. To determine the intervals RR, PR, QT, corrected QT (QTc), and QRS complex, a period of 10 s was analyzed in the ECG tracing of each animal. The QT interval was measured starting from the onset of the QRS complex until the end of the T wave, which is the return of the T wave to the baseline. QTc was obtained using Bazett's formula (QTc = QT/HRR) (17). Parameters were analyzed using previously described procedures (18).

### Statistical Analyses

Values were expressed as mean ±SEM. Statistical analyses were performed using Prism software. Repeated measures one-way ANOVA was used to test for differences in each parameter at 0, 3, and 5 weeks. For statistically significant parameters, *post-hoc* pairwise *t*-tests were conducted using Tukey's method for correcting for multiple comparisons. To assess the relationship between invasively measured RVSP and echocardiographic parameters, control and study animals were pooled, and Pearson correlations were calculated. Fisher's *z* transformation was used to calculate 95% confidence intervals. Due to the large number of tested parameters, a Bonferroni correction was applied to adjust for multiple comparisons.

## Results

### Invasive Hemodynamics

**Figure 1:**
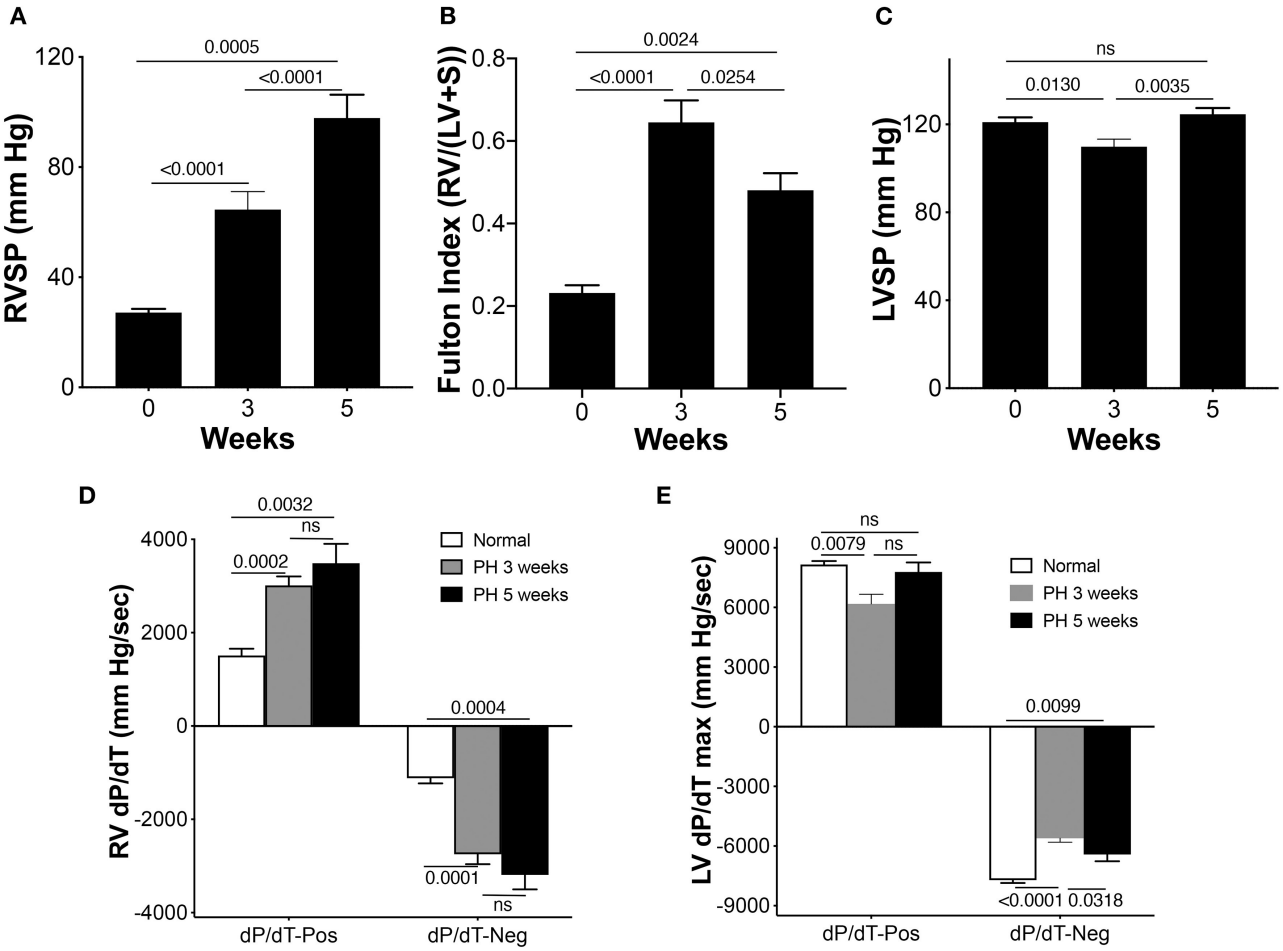
Right ventricle (RV) pressures and electrocardiographic parameters in pulmonary arterial hypertension (PAH) rats. Pulmonary arterial hypertension was induced in rats as described in the Methods section. **(A)** The RV systolic pressure (RVSP) was measured in separate sets of control animals and animals at 3 and 5 weeks during the development of PAH, *n* = 10–17 animals/group. **(B)** Fulton Index (*n* = 6–9 animals/group). **(C)** Left ventricle systolic pressure (LVSP), *n* = 10–17 animals/group. **(D)** Rates of rise and decline of the RV pressure in systole (above baseline) and in diastole (below baseline) (dP/dT) were recorded, *n* = 10–17 animals/group. **(E)** Rates of rise and decline of the LV pressure in systole (above baseline) and in diastole (below baseline) (dP/dT) were recorded, *n* = 9–17 animals/group.

A rat SuHyx model of PAH was used as described before (11). Right ventricular systolic pressure (RVSP) and hypertrophy were measured at 0, 3, and 5 weeks to confirm progression and establishment of PH. Figure 1A demonstrates a steady increase in RVSP in the PAH rats at 3 and 5 weeks, when compared with the controls. As expected in this model, the chronic increase in RV afterload led to RV hypertrophy shown by an increase in the Fulton index both at 3 and 5 weeks compared with controls (Figure 1B). Hypertrophy was more at the 3-week time point compared with the 5-week time point. LV systolic pressure (LVSP) remained unaltered in the PAH group at 5 weeks but was decreased at the 3-week time point (Figure 1C). The rate of rise of RV pressure during ejection and post ejection phases of the cardiac cycle was used to assess the contractile and relaxation properties of the RV. RV dP/dT_maximum_ was substantially elevated in the PAH animals at 3 and 5 weeks when compared with the controls (Figure 1D). Similarly, the RV dP/dT_minimum_ at 3 and 5 weeks were increased in the PAH animals when compared with the controls (Figure 1D). Since RV dysfunction can alter LV contractility, we measured the LV dP/dT. Interestingly, LV dP/dT positive did not differ from the control at 5 weeks, but there was a decrease at the 3-week time point (Figure 1E). However, the LV dP/dT negative in the PAH group at 3 and 5 weeks were both diminished when compared with the controls (Figure 1E).

### Electrocardiography

**Figure 2:**
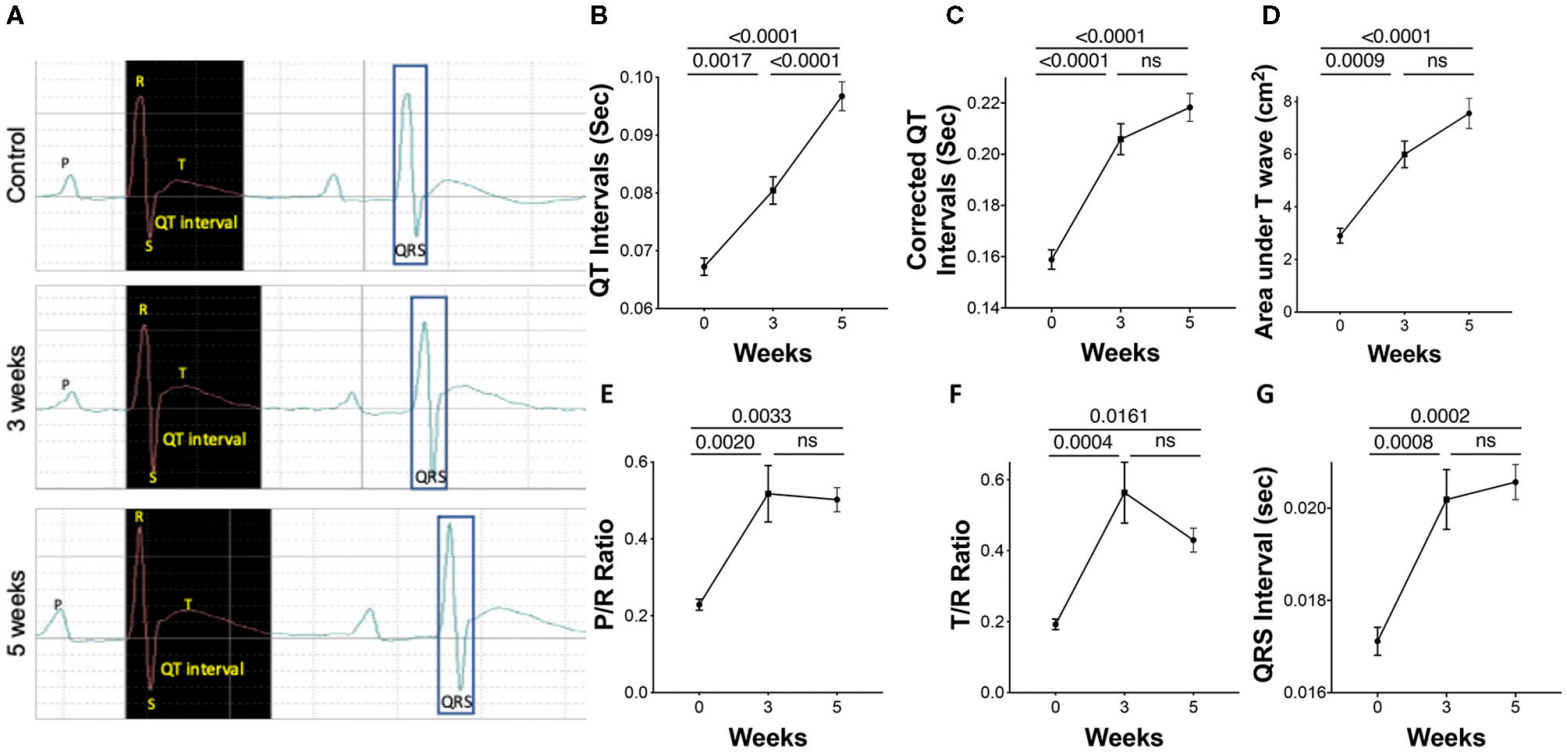
Electrocardiographic parameters during PAH progression in rats. Pulmonary arterial hypertension was induced in rats as described in the Methods. A 2-channel electrocardiography (EKG) was utilized to record parameters. **(A)** Representative EKG tracings, showing **(B)** uncorrected QT intervals, **(C)** corrected QT intervals, **(D)** area under the T wave, **(E)** P/R ratio, **(F)** T/R ratio, and **(G)** QRS interval was also performed. *N* = 7–10 animals/group.

Polarization characteristics of the heart chambers resulting from adaptation and maladaptation were assessed using electrocardiography (EKG). Representative tracing at all three time points shows changes with progression of PAH (Figure 2A). A significant prolongation in the corrected QT interval (QTc), an increase in the amplitude of P and T waves, and a widening of the QRS complex were observed in the PAH animals across the three time points, 0, 3, and 5 weeks (Figures 2B–G).

### Lung Histology and Cardiac Markers of Injury

**Figure 3:**
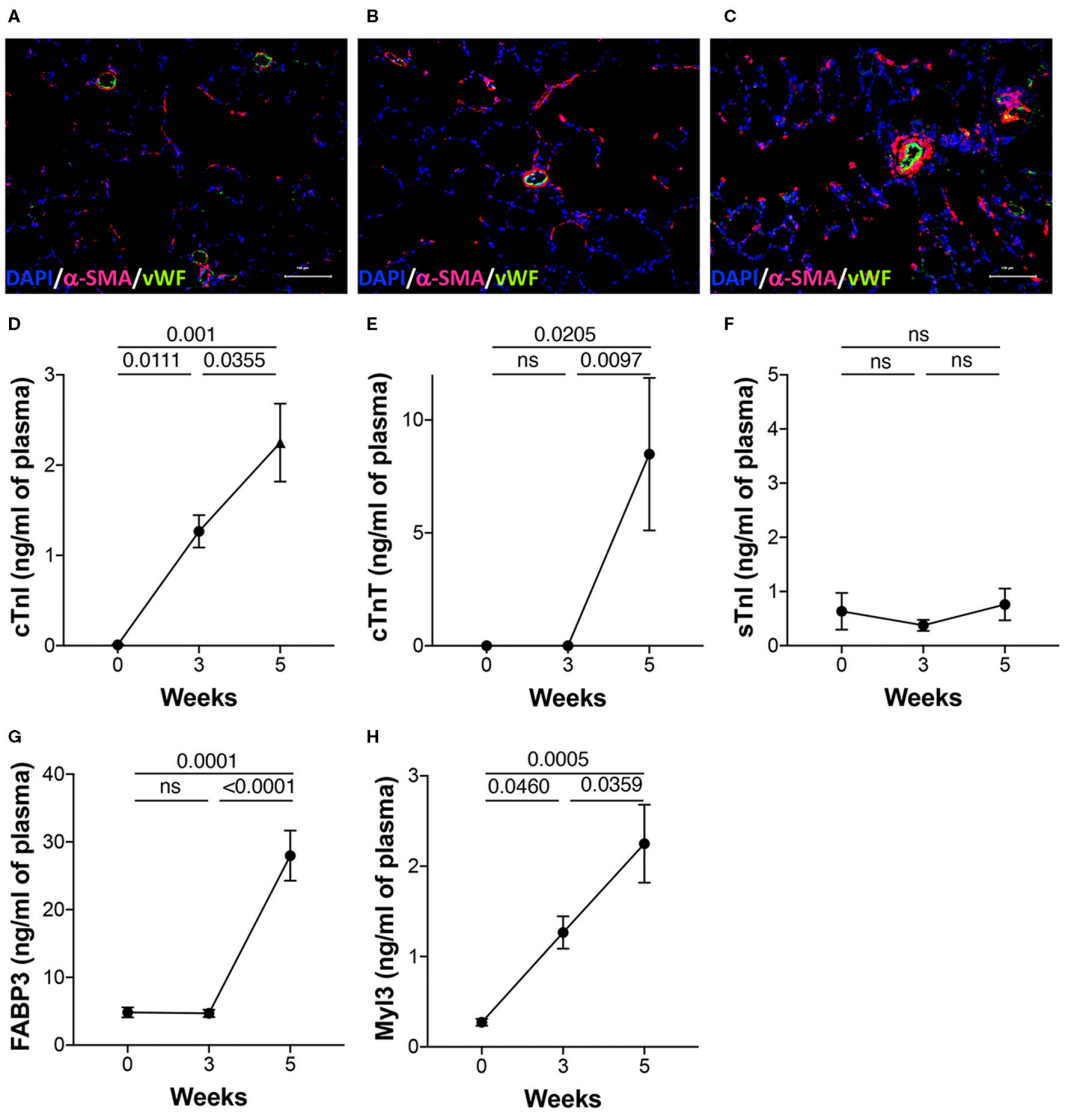
Lung histology and plasma biomarkers of cardiac injury. PAH was induced in rats over a 5-week period as described in the Methods. **(A–C)** Representative images of the lung sections from the three time points stained for anti-vWF and anti-αSMA as described in details in the Methods. DAPI was used as a counterstain to visualize nuclei in tissue. Plasma was collected from separate sets of animals from naïve (0 day), 3 and 5 weeks post-induction of PAH. Markers of injury were estimated in the plasma using a multiplexed, meso scale discovery platform for **(D)** cardiac troponin I (cTnI), **(E)** cardiac troponin T (cTnT), **(F)** skeletal troponin I (sTnI), **(G)** fatty acid binding protein 3 (FABP3), and **(H)** myosin light chain 3 (Myl3). *N* = 5–9 animals/group.

To validate PAH pathology, the lung sections were stained for vWF and α-SMA to highlight changes in the intima and media of the arteries, respectively, during disease progression. As expected, the lung histology showed increased muscularization of the arteries with time demonstrating the progressive nature of the disease in this model (Figures 3A–C). Markers of cardiac injury are known to increase in pulmonary hypertension. cTnI increased linearly with disease progression (Figure 3D). Similarly, myosin light chain 3 (Myl3), a ventricular and slow skeletal muscle isoform, also increased linearly with disease progression (Figure 3H). Cardiac troponin T, however, increased only at the 5-week time point (Figure 3E). As expected, sTnI did not change with disease severity (Figure 3F). FABP3 (aka: H-FABP; heart type fatty acid-binding protein) increased only at the 5-week time point (Figure 3G).

### Echocadiographic Estimation of Pulmonary Pressures and Pulmonary Vascular Resistance

**Figure 4:**
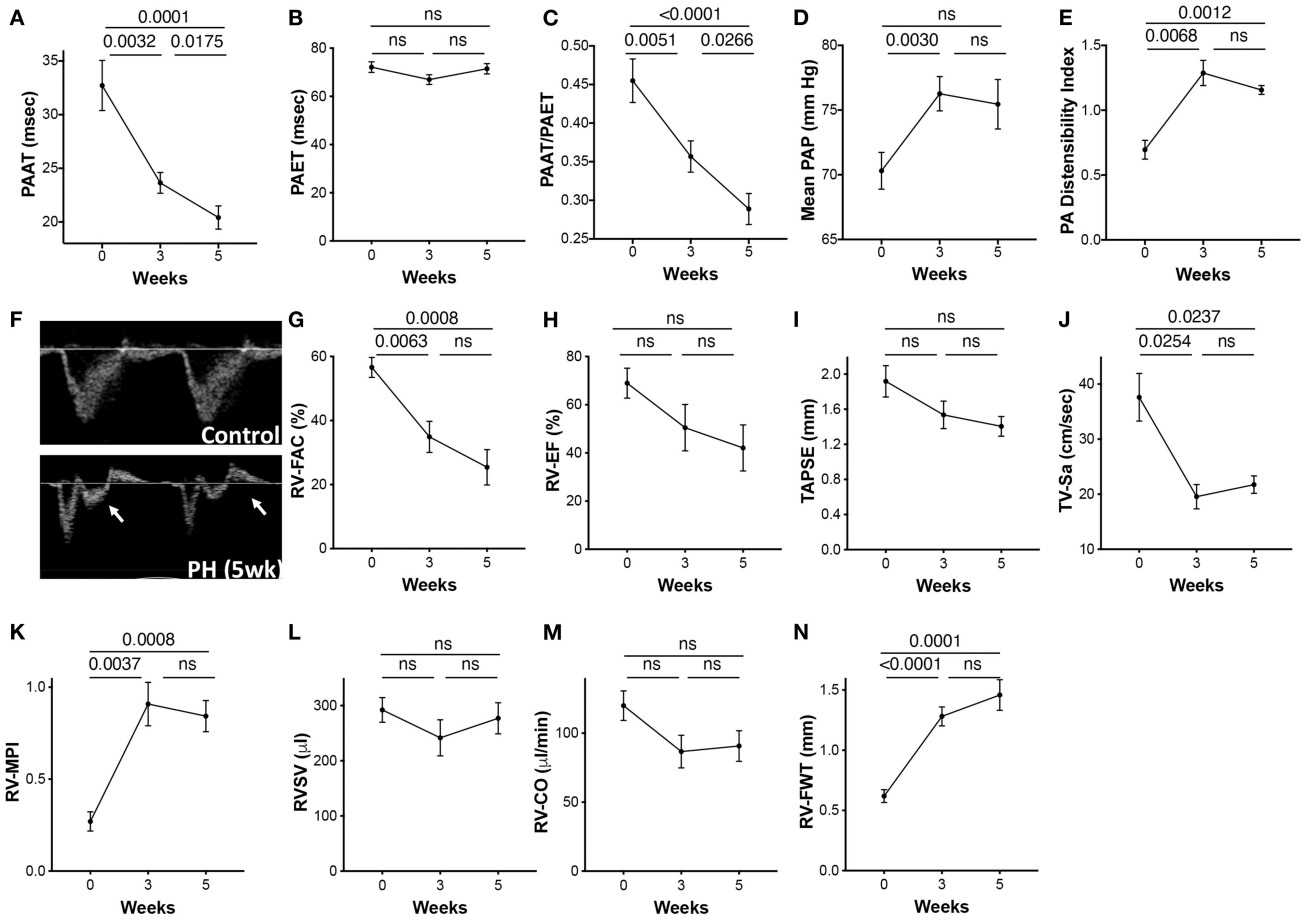
Echocardiographic parameters of the pulmonary artery and the RV during progression of PAH. PAH was induced in rats over a 5-week period as described in the Methods. Echocardiographic images were acquired prior to induction of PH (0 day) and at 3 and 5 weeks post-induction. **(A)** Pulmonary artery acceleration time (PAAT), **(B)** pulmonary artery ejection time (PAET), **(C)** PAAT/PAET ratio, **(D)** calculated mean pulmonary artery pressure (mPAP), and **(E)** PA distensibility index, calculated as the ratio of the PA diameter (obtained from parasternal RV outflow tract view) to ascending aortic diameter from parasternal long axis flow at end diastole. **(F)** Pulse wave Doppler tracings showing the PA flow obtained from parasternal RV outflow view. Arrow shows the PA mid systolic notch. Each animal served as its own control at previous time points. A 2-dimensional echocardiographic examination of the RV was also performed from multiple acoustic views. **(G)** RV fractional area of change (RV-FAC) was measured from the transgastric midpapillary view, **(H)** RV-EF, **(I)** Tricuspid annular plane systolic excursion (TAPSE), **(J)** Tricuspid peak annular systolic velocity (TV-Sa), and **(K)** RV myocardial performance index (RV-MPI). **(L)** RVSV and **(M)** RV-CO were calculated from the parasternal long axis view. **(N)** RV free wall thickness (FWT) was measured from the apical four-chamber views. *N* = 10 animals/group.

In addition to the invasive RVSP, we also measured non-invasive surrogates of PH using echo Doppler across three time points. PAAT and PAAT/PAET were reduced with disease progression (Figures 4A,C). A non-significant reduction in PAET from baseline to 3 weeks occurred (Figure 4B). Calculated values of mPAP using PAAT increased significantly at 3 weeks of PH. Although, mPAP was expected to increase with progression of PH, calculated values did not increase further between 3 and 5 weeks (Figure 4D). The PA diameter as assessed by echocardiography progressively increased across the three time points. The PA distensibility index was significantly increased at 3 weeks with no further increase at 5 weeks (Figure 4E). Increased PA resistance and a reduction in compliance of large PAs cause premature systolic PA wave reflection resulting in flow deceleration and a mid-systolic notch. A mid-systolic notch was discernable with progressive PAH at 5 weeks (Figure 4F).

### Right Ventricle Function and Structure

RV fractional area of change (RV-FAC) and ejection fraction (EF) reflect global RV systolic function. Tricuspid annular plane systolic excursion (TAPSE) and tricuspid valve systolic wave (TV-Sa) can serve as surrogates of the systolic function of the RV. Although, variable, RV systolic function was significantly reduced with progression of PAH. RV-FAC was significantly reduced at 3 weeks and continued to decline over 5 weeks (Figure 4G). Both EF and TAPSE, as measured by M-mode echocardiography tended to decrease with PH but were not statistically significant (Figures 4H,I). TV-Sa was significantly reduced starting at 3 weeks (Figure 4J). Similarly, RV myocardial performance index (RV-MPI), a measure of global systolic and diastolic RV function (19), was also increased at 3 weeks with no further change from 3 to 5 weeks (Figure 4K). Interestingly, RVSV did not change across the three time points (Figure 4L). However, a non-statistical reduction in RV cardiac output (RV-CO) at an early time point was observed (Figure 4M).

Increased RV pressure overload results in RV hypertrophy. The RV free wall thickness (RV-FWT), an indicator of RV hypertrophy, was significantly increased at 3 weeks. Furthermore, modest non-significant FWT changes occurred at 3- to 5-week time point (Figure 4N). An increase in FWT is consistent with increased Fulton index at 3 and 5 weeks. No significant change in the RV diastolic function (RV E/A ratio, E′, or E/E′) was noticed across the three time points (data not shown).

### Inferior Vena Cava and Hepatic Venous Flows

**Figure 5:**
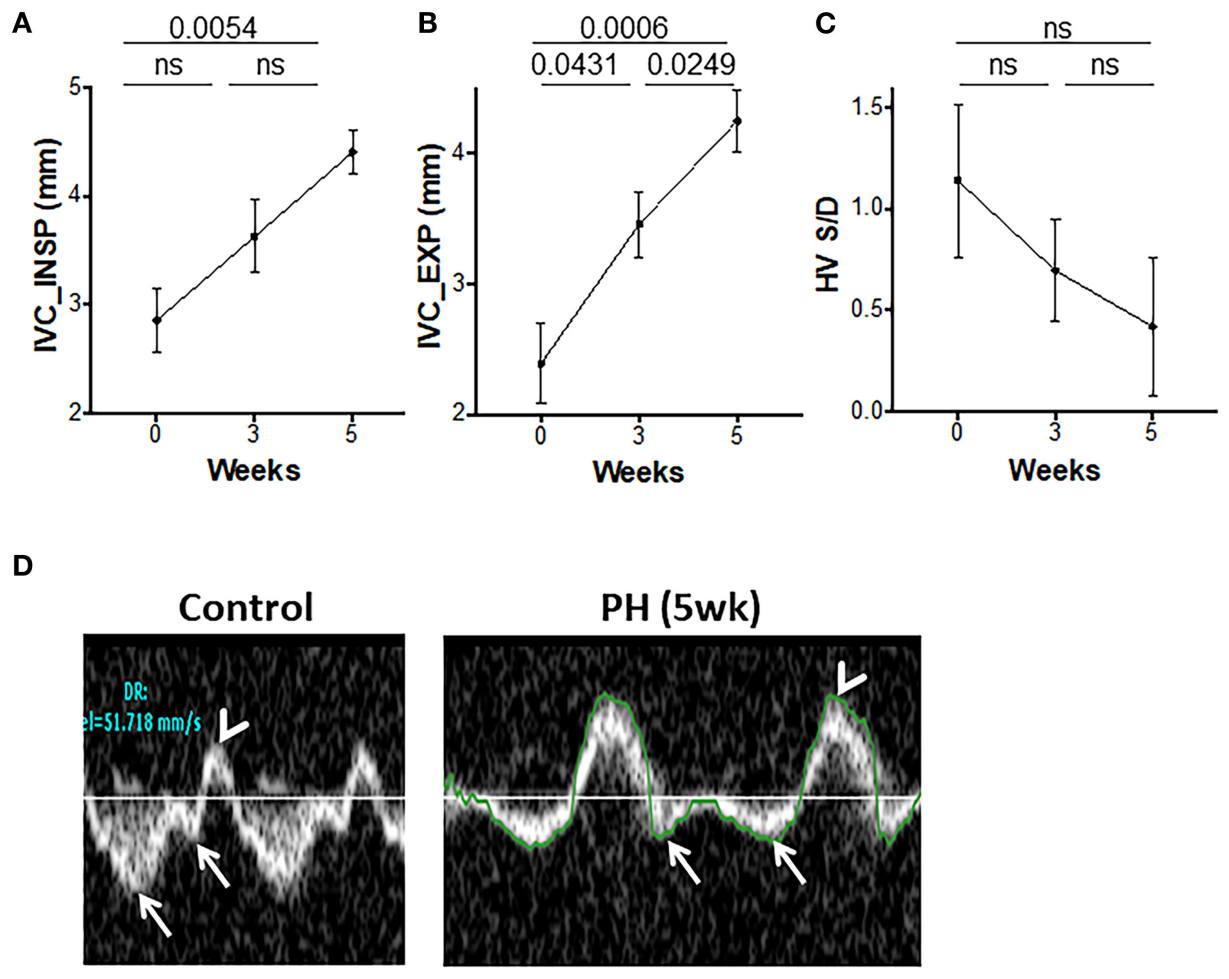
Inferior vena cava (IVC) and hepatic venous flows during progression of PAH. IVC diameters at end inspiration **(A)** and end expiration **(B)** and the ratio of hepatic venous systolic to diastolic peak velocities **(C)** were measured from the subcostal view. **(D)** Representative echocardiographic images showing hepatic venous flow pattern with a reversal of systolic to diastolic peak velocity ratio (arrows) and prominent atrial reversal peak velocity wave (arrowhead) in control and animals with PH. *N* = 10 animals/group.

The hepatic venous (HV) flows and IVC diameter indicate the flow upstream from the PA and can be altered in PAH. The IVC diameters progressively increased from 0 to 5 weeks (Figures 5A,B). An increase in RV-EDP can lead to an increase in the amplitude of the HV atrial reversal waveform. A non-significant reduction in the S/D over time was observed (Figure 5C). Peak velocity of the HV atrial reversal wave was also increased (Figure 5D).

### Left Ventricle Function and Structure

**Figure 6:**
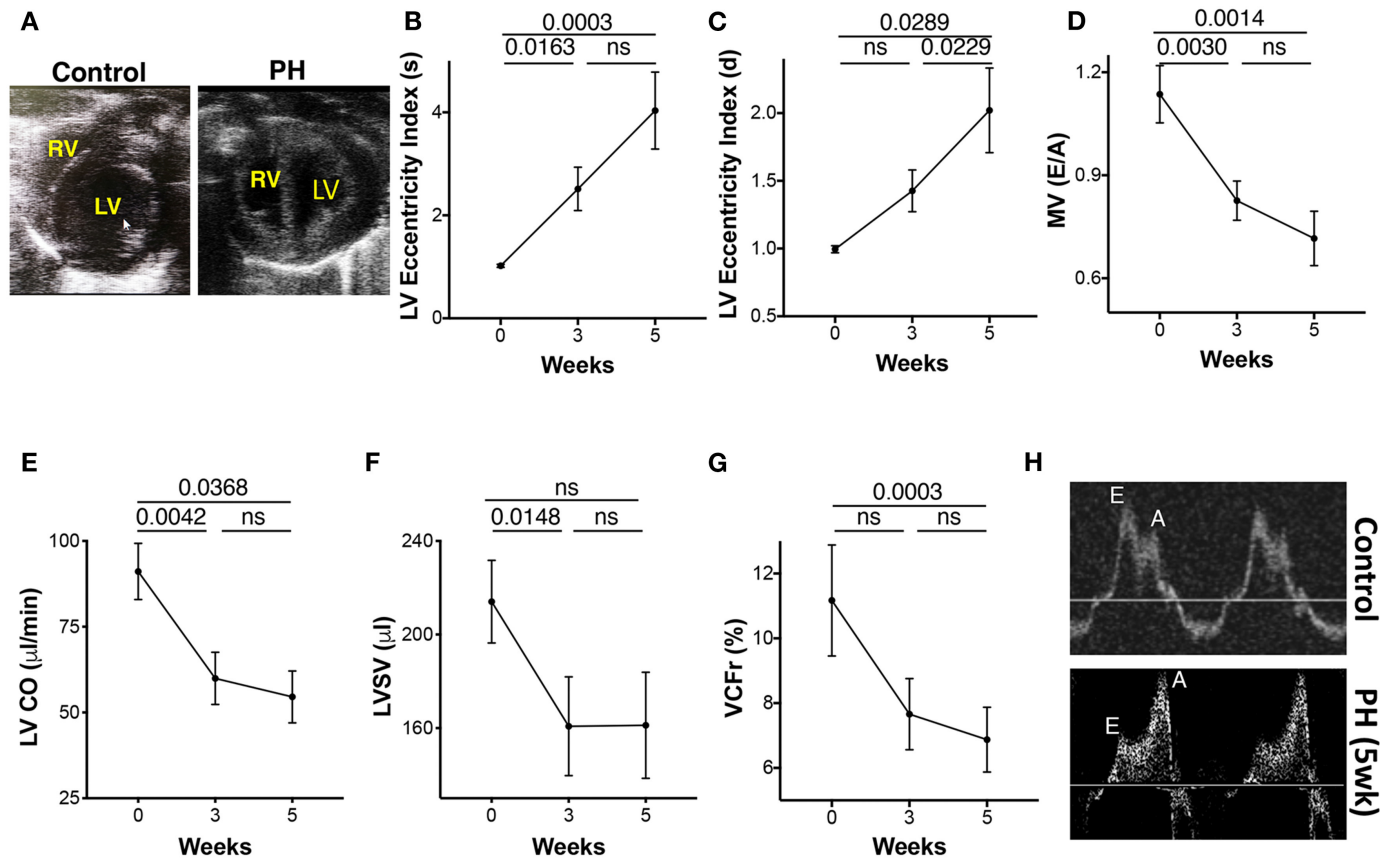
Echocardiographic parameters of the left ventricle (LV) during progression of PAH. **(A)** Apical view of the chambers showing the RV and LV. LV eccentricity index image showing a reduction in the septolateral diameter in relation to the antero-inferior diameter of the LV. Eccentricity index was calculated from the transgastric midpapillary short axis view as the ratio of the antero-inferior diameter to the septolateral diameter of the LV. **(B)** LV eccentricity index in systole, **(C)** LV eccentricity index in diastole, **(D)** Transmitral ratio of peak early to late diastolic wave velocity, **(E)** CO, **(F)** SV, and **(G)** LV velocity of circumferential shortening as measured from the parasternal long axis view at 0, 3, and 5 weeks of PH induction. **(H)** Echocardiographic tracings showing mitral valve flow in control (top) and PH (bottom) animals. *N* = 10 animals/group.

The progressive increase in RV afterload can compromise LV function, structure, and filling. The inter-ventricular septum was flattened with a leftward shift (Figure 6A). The LV eccentricity index systolic (LV-EIs) and the LV eccentricity index diastolic (LV-EId) increased progressively from baseline to 5 weeks (Figures 6B,C). A significant reduction in the LV filling was demonstrated by a reduced E and E/A from baseline to week 3, with a trend toward further reduction afterward (Figures 6D,H). LV cardiac output (LV-CO) was significantly reduced at week 3 with modest changes thereafter (Figure 6E). LV-SV was reduced at 3 weeks with no additional change with increased severity of PAH (Figure 6F). There was a significant reduction in the LV velocity of circumferential shortening (LV-VCFr) at 5 weeks (Figure 6G). In summary, reduced LV filling and output early in the disease was followed by reduction in the LV contractility at more advanced stage of PAH.

### Correlation Between Echocardiographic Variables and Invasively Measured Right Ventricular Systolic Pressure

Pooled analyses of the study and control animals revealed significant correlation between invasively measured RVSP and each of the following parameters: pulmonary artery acceleration time (PAAT), PAAT/PAET (pulmonary artery ejection time), IVC diameter, RV-FAC, TAPSE, LV-EIs, LV-CO, LV-SV, and trans-mitral E/A (Table 1).

**Table 1:** Correlation of echocardiographic variable with right ventricular systolic pressure (RVSP).

**Variable**	**Correlation coefficient**	***p*-Value**
HV D	−0.44	0.053
HV S/D	−0.45	0.044
HV S2	−0.06	0.792
IVC_INSP	0.31	0.185
IVC-EXP	0.56	0.010
LV CO	−0.65	0.002
LV eccentricity index d	0.52	0.027
LV eccentricity index s	0.58	0.013
MPAP (common)	−0.62	0.055
MPAP (PAAT <120 ms)	−0.39	0.271
MV (E/A)	−0.77	<0.001
PA distensibility index	0.56	0.045
PAAT	−0.63	0.004
PAET	0.26	0.266
PAAT/PAET	−0.64	0.003
RV-EF	−0.34	0.208
RV-FAC	−0.60	0.005
RV mass	0.12	0.671
RV-MPI	0.48	0.053
RV-CO	−0.51	0.075
RVSV	−0.52	0.066
LVSV	−0.58	0.007
TAPSE	−0.50	0.025
TV-Sa	−0.09	0.714

## Discussion

Using serial measurements, we identified distinct patterns of EKG, and biochemical and echocardiographic parameters that together can potentially be used to detect PAH early, monitor PAH progression, and assess RV dysfunction and its response to treatment. Knowledge of these patterns addresses the current gap in practice that focuses primarily on PAP reduction rather than on holistically reversing myocardial and vascular remodeling (2). Echocardiographic markers showed three different patterns representative of the pulmonary vascular and cardiac remodeling that takes place during the course of PAH; a steady pattern in which there is a progressive change of the echo parameters across the three time points of PAH (IVC end inspiratory, IVC end expiratory diameter, and PAAT), an early change in which there is an early reduction [RV-FAC, TV-Sa, MV(E/A), LV-CO, LVSV] or increase (mPAP by PAAT, RV-MPI, LV-EIs) followed by a plateau at severe PH, and a late pattern in which there is only a late rise at severe stages of PAH (LV-EId). In the same animal model, plasma biomarkers of cardiac injury showed two different patterns. Plasma levels of Myl-3 and cTnI steadily increased across the three time points compared with FABP-3 and cTnT that showed only a late rise at the 5-week time point. Grouping these variables into “patterns” overcomes some of their individual limitations in terms of their temporal relationship to the severity and progression of PAH.

PAH is characterized by a decrease in pulmonary vascular compliance and an increase in PVR causing initial adaptive compensation followed by a maladaptive decompensatory phase of the RV failure (20). The patterns identified in our study capture some of these mechanisms. The presence of mid-systolic notching on the RV outflow tract spectral Doppler observed at the 5-week time point can be used as a qualitative marker of the reduction in the PA compliance responsible for the increase in pulsatile load of the RV, which precedes the increase in PVR. It can also be used as a qualitative surrogate of the RV/PA uncoupling, an important measure in determining the RV maladaptation in PAH (21).

With progressive increase in mPAP, less time is spent during ejection, and a faster rise in peak systolic pressure occurs due to a rapid closure of the pulmonary valve, causing reduction in PAAT and PAAT/PAET ratio (22). The reduction in PAAT is indicative of increased PVR and is a consistent finding in preclinical and clinical PH, further supporting its reliability in monitoring the disease progression (22). Although, there has been some success in echocardiographically estimated mPAP, consistent determination of estimates remain a challenge (23, 24).

RV hypertrophy is the hallmark of PAH, and an increase in the RV free wall thickness (FWT) signifies an important compensatory mechanism by which the RV reduces its wall stress induced by the increase in pressure overload. Here we used the RV-FWT as a surrogate of RV hypertrophy rather than measuring the RV mass, given the limitations of measuring the RV mass by M-mode echocardiography (14). These findings were confirmed by Fulton index measurements and are consistent with other reports (25) and may represent a compensatory mechanism. The RV contractility as measured by dP/dT_max_ increased significantly during the early phase of PAH development and consistent with other reports (26, 27). However, changes from 3 to 5 weeks were not significant despite a significant increase in RVSP and may represent the maladaptive phase of PAH. High RV dP/dT_max_ positive values were observed in PH patients even with evidence of RV failure (28). In advanced stages of PAH, dP/dT may be more dependent on the RV mass, HR, and intracavitary pressure rather than myocardial contractility (29).

Increased PA pressures can alter hepatic venous (HV) flows and IVC diameter leading to a reduction in the forward flow from the HV to the RV. An increase in IVC diameter reflects an increase in back pressure from the right atrium as a result of increase in RV afterload and can serve as a prognostic indicator of PH (30). Hepatic venous flow was shown to plateau after 3 weeks reflecting sensitivity only during the initial phases of development of PAH. This reflects the variable presentation of PAH in terms of the development of RV failure, elevation of the RV-EDP, occurrence of significant TR, and occurrence of atrial fibrillation (AF). Although, TR is a consistent finding in humans with PH, we were unable to discern a consistent tricuspid regurgitant jet due to technical limitations in image acquisition in rats.

We analyzed the correlation between the echocardiographic markers of progression of PAH and RVSP in order to assess their association with the severity of PAH. Markers associated with severity of PAH may be used for prognostication, whereas those associated with progression of PAH may be sought for monitoring of the disease progression and response to therapy. The correlation of TAPSE with RVSP confirms its prognostic significance and is consistent with previous reports. However, its failure to reduce beyond 3 weeks of PAH progression can be explained by the RV assuming a more spherical configuration during advanced stages of PAH and that TAPSE, being representative of the longitudinal motion of the RV and only of the free wall of the TV annulus, may be less contributing to RV ejection at this advanced stage of PAH. We are aware of the limitations of M-mode echocardiography in measuring RVEF compared with 3D echocardiography, which we did not possess at the time of the study. Similarly, RV-FAC, while being a prognostic marker of PAH, may not be an ideal marker of progression since it lacks representation of the RV outflow tract and may not represent the intrinsic contractility of the RV. Overall, echocardiography is a valuable tool in monitoring the severity and progression of PAH and may be helpful in its early diagnosis.

In the model, serial changes in the LV with progressive PAH were also characterized. The rise in LVSP pattern is consistent with previous reports (31). Flattening of the inter-ventricular septum with a leftward shift is also consistent with other studies (32). This reduces LV septo-lateral dimension compared with antero-inferior dimension at end systole and diastole reflecting ventricular interdependence (33). The initial pattern of reduced filling is evidenced by a decrease in transmitral E/A and an increase in LV-EIs and LV-EId, followed by a reduction in LV-CO and LV-SV, and ending with a reduction in contractility as shown by reduced VCFr. The latter is considered a less load-dependent index of systolic function compared with LV-EF (34).

In our EKG studies, progressive prolongation in QT-interval reflects an abnormality in ventricular depolarization or repolarization, which may predispose to ventricular arrhythmias reportedly common in PH (35). Prolongation of QT-interval has been shown to correlate with cardiac remodeling, in addition to being an independent predictor of mortality in PH (36). Increased QT-interval and wide RS-interval observed in our studies were also consistent with other reports where QRS prolongation was associated with clinical severity and mortality in patients with PH (37, 38). An increase in the QRS interval is a sign of intraventricular electrical conduction delay, likely due to ventricular dys-synchrony resulting from RV hypertrophy and dilation. Increased amplitude of the P wave is a sign of atrial enlargement likely related to right atrial enlargement secondary to elevated RVSP and secondary to PH (18). These EKG findings of raised right atrial pressure, intraventricular conduction delay, and propensity for ventricular arrhythmias are consistent with adverse outcomes in PH (39).

Makers of cardiac injury are frequently used in stratification of disease. Our findings of increased levels of cTnT and FABP3 only during the late stages of PAH suggests their potential use as markers of disease severity. It is therefore not surprising that both of these markers were shown to correlate with major adverse events and were also predictors of mortality (40, 41). On the other hand, cTnI levels increased linearly with PAH progression suggesting its potential use as a sensitive marker of disease progression and in response to therapies. We also found that Myl3, another marker of cardiotoxicity (42, 43), also increased with disease severity and can also be potentially used as a sensitive marker of PH progression.

In summary, recognizing biochemical, electrocardiographic, and echocardiographic patterns of PAH progression and severity may help in the monitoring and prognostication of RV function in PAH. Despite limitations, echocardiography is invaluable not only in diagnosing PAH but also in follow-up. There is a need for a “collective” assessment of the entire cardiovascular system in PAH. More studies are needed to mechanistically correlate electrical, vascular, and mechanical remodeling to non-invasive echo- and electrocardiographic findings.

## Data Availability Statement

The original contributions presented in the study are included in the article/supplementary material, further inquiries can be directed to the corresponding author/s.

## Ethics Statement

The animal study was reviewed and approved by Institutional Animal Care and Use Committee (IACUC), University of Alabama at Birmingham, Birmingham, Alabama, USA.

## Author Contributions

AZ and AA helped conceive the idea, design the study, analyze the data, and write the manuscript. IZ, MH, and NM helped acquire and analyze the data and review and write the manuscript. JM-J and TH helped analyze the data and review and write the manuscript. CM and MF helped with the statistical analysis of the data and reviewed and edited the manuscript. All authors contributed to the article and approved the submitted version.

## Conflict of Interest

The authors declare that the research was conducted in the absence of any commercial or financial relationships that could be construed as a potential conflict of interest.

## Abbreviations

HV D: hepatic vein diastolic
HV S/D: hepatic vein systolic/diastolic
IVC_EXP: inferior vena cava expiratory diameter
IVC_INSP: inferior vena cava inspiratory diameter
LV CO: left ventricle cardiac output
LVSV: left ventricle stroke volume
LV-EIs: left ventricle eccentricity index systolic
LV-Eid: left ventricle eccentricity index diastolic
LV-VCFr: left ventricle circumferential shortening
MPAP: mean pulmonary artery pressure
MV(E/A): early diastolic mitral velocity/late diastolic mitral velocity
PAAT: pulmonary artery acceleration time
PAET: pulmonary artery ejection time
RVEDV: right ventricle end diastolic volume
RVESV: right ventricle end systolic volume
RVEDP: right ventricle end diastolic pressure
RVESP: right ventricle end systolic pressure
RV-EF: right ventricle ejection fraction
RV-FAC: right ventricle fractional area change
RV-FWT: right ventricle free wall thickness
RV-MPI: right ventricle myocardial performance index
RV-CO: right ventricle cardiac output
RVSV: right ventricle stroke volume
TAPSE: tricuspid annular plane systolic excursion
TV-Sa: tricuspid valve systolic wave.
